# Associations of midlife fitness and obesity profiles with cognitive function

**DOI:** 10.1002/ejsc.12067

**Published:** 2024-03-18

**Authors:** Jamie L. Tait, Taya A. Collyer, Seana L. Gall, Alison J. Venn, Terence Dwyer, Brooklyn J. Fraser, Chris Moran, Velandai K. Srikanth, Michele L. Callisaya

**Affiliations:** ^1^ Institute for Physical Activity and Nutrition School of Exercise and Nutrition Sciences Deakin University Geelong Victoria Australia; ^2^ National Centre for Healthy Ageing Melbourne Victoria Australia; ^3^ Peninsula Clinical School Central Clinical School Monash University Frankston Victoria Australia; ^4^ Menzies Institute for Medical Research University of Tasmania Hobart Tasmania Australia; ^5^ The School of Clinical Sciences Monash Medical Centre Clayton Victoria Australia; ^6^ The Nuffield Department of Women's & Reproductive Health University of Oxford Oxford UK; ^7^ Murdoch Children's Research Institute Melbourne Victoria Australia; ^8^ Faculty of Medicine Dentistry and Health Sciences University of Melbourne Melbourne Victoria Australia; ^9^ School of Public Health and Preventive Medicine Monash University Melbourne Victoria Australia

**Keywords:** epidemiology, physical fitness, psychomotor, strength

## Abstract

Low fitness and obesity at midlife are individually associated with later‐life decline in cognition and health. However, the relationship between profiles of midlife fitness and obesity with midlife cognition is unclear and was examined by this study. Participants from the third follow‐up of the Childhood Determinants of Adult Health study (2014–2019; aged 36–49 years) were assessed for obesity (body mass index [BMI]), cardiorespiratory (physical work capacity) and muscular fitness (isometric grip and leg strength), and these factors were entered into a latent profile analysis. Psychomotor‐attention, learning–working memory and global cognition composites were derived from *z*‐scores of individual CogState battery tests. Linear regression analyses examined associations between profiles of fitness and obesity with cognition, adjusting for age, sex, education, alcohol and smoking. Using data from 617 participants [44.4 ± 2.6 (mean ± SD) years, 52% female], we identified 5 mutually exclusive profiles of obesity and fitness. Relative to those with the lowest fitness and highest BMI (5% of sample), those with a profile of (1) highest muscular fitness and average BMI (9%), (2) average muscular fitness and BMI (61%) and (3) highest cardiorespiratory fitness (CRF) and below average BMI (7%) all exhibited better global cognition [at least 0.44 (0.12, 0.77) SD, 95% CI] and psychomotor‐attention performance [at least 0.59 (0.27, 0.91) SD, 95% CI]. Compared with low fitness and unhealthy BMI levels, possessing average or high muscular and CRF and a healthy BMI in midlife was associated with better cognitive performance. Enhancing fitness and reducing obesity in midlife could contribute to improved cognitive performance in middle age.

## Highlights

- Profiles containing adults with a combination of low levels of cardiorespiratory and muscular fitness and higher levels of obesity were associated with lower psychomotor‐attention and global cognition at midlife.
- Possessing average or higher levels of fitness, and lower obesity at midlife, was associated with better cognitive performance at midlife compared to those with lower fitness and unhealthy BMIs.
- Maintaining fitness and reducing obesity at midlife may contribute to greater cognitive performance at midlife and beyond and reduce the risk of cognitive decline.

## 1 INTRODUCTION

Cognitive decline and dementia are public health concerns for which there is no cure. Cognitive performance can start to decline as early as midlife (Rovio et al., 2016), and lower cognition in midlife is associated with a greater likelihood of developing mild cognitive impairment and dementia in older age (Knopman et al., 2018). Therefore, it is important to understand the factors associated with cognition earlier in the life course in order to develop effective preventative strategies.

Poor physical function such as weaker muscular strength, lower cardiorespiratory fitness (CRF) and slower gait speed has been associated with worse cognitive performance in older adults (Clouston et al., 2013; Cui et al., 2021; Mekari et al., 2019). Furthermore, in longitudinal studies of middle‐aged and older adults, stronger grip strength and higher CRF are associated with slower cognitive decline (Liu et al., 2019) and reduced risk of dementia in later life (Cui et al., 2021; DeFina et al., 2013). Although there are fewer studies in middle age (Adamo et al., 2020; Malmstrom et al., 2005), similar associations have been demonstrated. For example, in middle age, weaker grip strength was cross‐sectionally associated with lower executive function, global cognition and verbal fluency (Adamo et al., 2020; Malmstrom et al., 2005), and lower CRF was associated with lower global cognition and memory (Tarumi et al., 2013). The mechanisms underpinning these relationships are uncertain. However, higher CRF or muscular strength in midlife may preserve cognition (Pedersen et al., 2012) through a reduction in cardiovascular risk factors, such as diabetes, that are associated with poorer cognition or through a direct effect on the brain via the promotion of neurogenesis and angiogenesis (Brown et al., 2013). Alternatively, fitness measures and cognitive processing may have similar underlying brain networks, where deterioration in these networks results in poorer performance in both cognition and fitness (Carson, 2018; Peng et al., 2020). Obesity at midlife has also been linked to poorer performance in verbal abilities, perceptual speed and memory at midlife (Dahl et al., 2013), as well as cognitive decline (Dahl et al., 2013) and dementia (Smith et al., 2011) in older age. This relationship is likely driven by vascular disease (Kivipelto et al., 2005), physical inactivity and pro‐inflammatory markers released from adipose tissue (Craft, 2009). However, collectively, these studies have mainly considered strength, obesity and CRF independently (Adamo et al., 2020; Dahl et al., 2013; Malmstrom et al., 2005; Tarumi et al., 2013) and included small sample sizes (*n* < 60) (Adamo et al., 2020; Tarumi et al., 2013).

Importantly, few studies have examined different profiles (subpopulations within a population based on a certain set of characteristic patterns) of fitness and obesity in relation to midlife cognition. Identifying distinct groups of individuals based on multiple factors may provide insights as to how these factors interact with each other and reduce the influence of collinearity that is seen with orthogonal models. Profiles, including low muscular strength and low CRF at childhood (Fraser et al., 2016) and obesity and low muscular strength at midlife (Lopez‐Lopez et al., 2021), have been previously associated with worse cardiovascular and metabolic health in midlife compared to healthier profiles. Given this, it is plausible that such phenotypic profiles may also be associated with poorer cognitive function in midlife. This is currently untested but may add value in providing a more nuanced approach to identifying groups of at risk adults at midlife for specific interventions to maintain brain health later in life. Therefore, the aim of this study was to examine the association between specific profiles of midlife fitness (physical work capacity, grip and leg strength) and obesity (BMI) and three measures of cognitive performance (e.g., global cognition, working memory and psychomotor‐attention). It was hypothesized that profiles with higher levels of obesity and lower fitness would be associated with poorer cognitive performance compared to profiles with lower obesity and higher fitness.

## 2 MATERIALS AND METHODS

This study incorporated a cross‐sectional analysis and was reported using STROBE guidelines. The 1985 Australian Schools Health and Fitness Survey was a nationally representative sample of 8498 Australian school children aged 7–15 years (Dwyer et al., 1994). The current analysis includes participants in the third follow‐up of this study: Childhood Determinants of Adult Health study (CDAH)‐3 who completed questionnaires and attended clinics between 2014 and 2019 (ages 36–49; *n* = 1567). Only those with a complete set of fitness and obesity measures and at least one measure of cognition were included (*n* = 617). At CDAH‐3, all participants gave written informed consent, and the Southern Tasmania Health and Medical Human Research Ethics Committee approved follow‐up studies.

### 2.1 Fitness measures

CRF was estimated sub‐maximally as physical working capacity at a heart rate of 170 beats per minute (PWC170) on a friction‐braked Monark 928G3r bicycle ergometer (Monark Exercise AB, Vansbro, Sweden) (Fraser et al., 2016). The sub‐maximal PWC170 test incorporated three successive 4‐min workloads that increased resistance stepwise at submaximal workloads, where participants were instructed to cycle at a constant 60 rpm. Workloads were selected on an individual basis to induce steady‐state heart rate responses at the end of each workload. Heart rate and power output (W) were recorded during the final minute of each workload using a Polar heart rate monitor (model RS300X; Polar Electro Oy, Kempele, Finland). These data were plotted, and the line of best fit was extrapolated to a heart rate of 170 beats per min to estimate physical work capacity at 170 beats per minute. PWC170 work rate was adjusted for lean body mass and expressed as watts per kg (W/kg) of lean body mass, calculated using previous methods (Fraser et al., 2016).

Muscular strength (grip and leg) was assessed using separate hand and leg–back isometric dynamometers (Smedley's Dynamometer, TTM, Tokyo, Japan). All measures of muscular strength were assessed twice, and the maximum attempt was included in analysis; hand grip was assessed before leg strength. Grip strength was assessed as the maximum voluntary contractile force of the right and left grip using an isometric hand dynamometer, which was adjusted to fit the participant's hand size. Participants started with the dominant hand, followed by the non‐dominant hand. Participants held a hand dynamometer with one hand, rested it on their opposite shoulder for stability and were asked to grip the dynamometer as hard as possible to the count of three and verbal encouragement was provided throughout. Instructions were to have at least 1 min between attempts on the same hand. The result from the dominant hand was used in analysis. For isometric leg strength, the technician demonstrated the test to the participants and they were provided with detailed instructions. Participants were then asked to stand flat footed on a leg‐back dynamometer with their back flat against a wall behind them. While holding a hand bar with an overhand grip, the participant flexed the knees until an angle of 115° was obtained and verified using a wooden 115° angle measure. At this point, a research technician attached the bar to the dynamometer by a chain. Participants then pulled upward on the bar as far as possible in attempting to extend their knees and hips against the bar. The participant practiced once, then the test was completed twice with at least 1 min between tests. During the test, the participant was asked to lift as hard as possible to the count of three and given verbal encouragement.

### 2.2 Obesity

Height was measured to the nearest 0.1 cm with a stadiometer (Invicta, Leicester, UK) and body weight was measured to the nearest 0.1 kg using a metric scale. Participants were instructed to wear light clothing suitable for doing the physical fitness tests; however, shoes, socks and bulky clothing were removed for measurement of body weight. Body mass index (BMI) was calculated [weight (kg)/height (m^2^)]. Using a constant tension tape, waist circumference was measured to the nearest 0.1 cm at the narrowest point between the lower costal border and the iliac crest in adulthood. Fitness and obesity measures were standardized by sex using the mean and standard deviation of the full cohort.

### 2.3 Cognition

Cognitive function was assessed using the CogState Brief Battery (CogState Ltd, Melbourne, Australia) using four tests: (1) A detection task test measuring simple reaction time (ms); (2) An identification task measuring choice reaction time (ms); (3). A One Card Learning task measuring visual memory (correct responses) and (4) A One Back task measuring working memory (ms). As previously reported and recommended by CogState (Maruff et al., 2013), data distributions from these tests were normalized. The reaction time scores of identification, detection and One Back were log10 transformed, while the square root of the proportion of correct responses on the One Card Learning task was arcsine transformed. CogState has been validated and is reliable in a range of participant age groups and clinical populations (Darby et al., 2014). Further detail on each task is presented in Table S1. Performances failing task criteria were removed. The One Card Learning task was not presented to participants at some clinics, resulting in fewer tests (*n* = 484/617). *Z*‐scores were calculated using the mean and standard deviation of the total sample for each task. Composite domains were then created as follows with higher scores indicating better performance (Lim et al., 2015):

- Global cognitive function: average for all tests
- Learning‐Working memory: average for One Card Learning and One Back Test
- Psychomotor‐Attention: average for Detection Task and Identification Task

### 2.4 Covariates

Questionnaires were used to collect age, sex and demographic data at follow‐up testing. Health conditions were self‐reported via questionnaire (e.g., cardiovascular disease and diabetes), while depression and anxiety during the lifetime were assessed via computerized Composite International Diagnostic Interview (CIDI‐Auto) (World Health Organization, 1997). Participants reported their highest level of education (ranging from secondary school to higher degree) and smoking status (current, ex or non‐smoker). Typical adult alcohol consumption was estimated from a food frequency questionnaire and converted to total intake per week (in grams) for analysis.

### 2.5 Statistical analysis

Participant characteristics were summarized using mean and SD or proportion and percentage. Characteristics between participants included in analysis and those not included were compared using *t*‐tests (continuous) and chi‐squared tests (categorical).

#### 2.5.1 Associations between fitness and obesity profiles and cognitive function

Latent profile analysis (LPA) was used to identify midlife fitness and obesity profiles. Given the involvement of multiple factors, it is difficult to employ a single hypothesis‐based approach to comprehend the connections between exposures. Data‐driven techniques may be more advantageous in this regard. LPA identifies mutually exclusive classes that display characteristic patterns across included variables by maximizing between‐group variance and minimizing within‐group variance (Oberski, 2016). Latent profiles therefore allocate each participant to the profile most likely given their fitness and BMI data. We estimated models with 2–10 profiles and used the Akaike and Bayesian Information Criteria, a scree‐plot of each model's log‐likelihood and clinical interpretability, to decide which model best fit the data (process shown in Figure S1). The LPA included PWC170 (CRF), grip strength (STR‐GRIP), leg strength (STR‐LEG), BMI and sex. Associations between resulting profile membership and each midlife cognitive composite were assessed using linear regression analysis. Model 1 was unadjusted, model 2 was adjusted for age, sex and education level and model 3 was additionally adjusted for midlife alcohol and smoking status after imputing missing data (*n* = 5 for alcohol and *n* = 36 for smoking) using chained equations (Lee et al., 2010). We did not adjust for medical history, such as hypertension and diabetes, as these variables were considered on the pathway between the fitness/obesity measures and cognition. Pregnant women were excluded from regression analyses as pregnancy is associated with lower cognitive function (Davies et al., 2018).

#### 2.5.2 Associations between fitness and obesity with cognition using stepwise regression

As secondary analyses, we also examined individual associations between standardized scores for each variable (BMI, CRF and muscular fitness scores), and each cognitive composite score using linear regression analysis adjusted for age, sex and education level (Model 1), and additionally for alcohol consumption and smoking at midlife (Model 2). Forward stepwise regression was then used to determine the significant predictors of each cognitive performance measure by systematically adding each fitness and obesity variable (e.g., BMI, then leg strength, grip and CRF) and adjusting for the covariates above. An orthogonal approach was used to analyze the individual effects of each factor in contrast to the profile approach. Residual plots were inspected for normality prior to analysis and the assumption of homoscedasticity verified. The significance level was set at *p* < 0.05. Statistical analyses were conducted using STATA 15.1 (STATA, College Station, TX, USA).

## 3 RESULTS

Table 1 shows the characteristics of participants included in the analysis (*n* = 617) and those not included (i.e., did not have complete set of fitness and obesity measures and did not complete cognitive testing; *n* = 950). The flow of participants through the study and reasons for non‐inclusion are presented in Figure S2. These reasons included time constraints, participant refused testing, injury, or occurrence of medical episode during testing. Characteristics between those included (*n* = 617) and not included (*n* = 950) were similar except that those who were included in the analysis were significantly older, had lower BMI and were less likely to be smokers or have hypertension. These differences were clinically small.

**TABLE 1:** Participant characteristics comparing those with a complete set of fitness and obesity measures and at least one measure of cognition (*n* = 617) to those who were not included (*n* = 950).

	*n*	All	Males	Females	*n*	Not included
*N*, %	617				950	
Age (years), mean (SD)		44.6 (2.5)	44.6 (2.5)	44.5 (2.6)		43.6 (3.0)
Females, *n* (%)		314 (50.9)				529 (55.6)
Weight (kg)	617	79.6 (16.4)	87.3 (14.0)	72.1 (15.1)	946	81.7 (18.9)
Height (cm)	617	172.5 (9.5)	179.7 (6.6)	165.6 (6.1)	950	171.9 (9.2)
Body Mass Index (kg/m^2^)	617	26.6 (4.7)	27.0 (4.0)	26.2 (5.2)	940	27.5 (5.7)
Grip strength (kg)	617	38.5 (11.3)	48.0 (7.2)	29.4 (5.4)	548	38.2 (10.9)
Leg strength (kg)	617	133.7 (49.8)	172.2 (37.1)	95.6 (26.9)	480	133.0 (45.7)
CRF, PWC_170_ (W/kg)	617	2.6 (1.0)	3.1 (1.1)	2.2 (0.7)	245	2.5 (0.8)
Waist‐to‐hip ratio	617	0.84 (0.08)	0.89 (0.06)	0.78 (0.07)	938	0.85 (0.09)
Global cognition z‐score	617	0.01 (−0.05, 0.06)	0.05 (−0.04, 0.14)	−0.03 (−0.11, 0.05)	627	−0.05 (−0.12, 0.01)
Learning‐working memoryz‐score	484	−0.02 (−0.09, 0.06)	0.06 (−0.04, 0.17)	−0.10 (−0.21, 0.01)	515	0.01 (−0.07, 0.08)
Psychomotor‐attention z‐score	613	0.03 (−0.04, 0.10)	0.07 (−0.04, 0.17)	−0.004 (−0.10, 0.09)	625	−0.08 (−0.16, −0.01)
Education, *n* (%)	617				931	
University education		340 (55.1)	156 (51.5)	184 (58.6)		485 (52.1)
Undergraduate diploma, vocational training		209 (33.9)	111 (36.6)	98 (31.2)		311 (33.4)
High school or less		68 (11.0)	36 (11.9)	32 (10.2)		135 (14.5)
Smoking status, *n* (%)	581				893	
Non smoker		385 (66.3)	190 (65.7)	195 (66.8)		545 (61.0)
Ex‐smoker		156 (26.9)	78 (27.0)	78 (26.7)		256 (28.7)
Smoker		40 (6.9)	21 (7.3)	19 (6.5)		92 (10.3)
Alcohol consumption (gm/week)	612	65.8 (90.3)	77.5 (102.1)	54.4 (75.7)	933	62.1 (115.0)
Medical history, *n* (%):						
Hypertension	613	67 (10.9)	38 (12.6)	29 (9.3)	940	167 (17.8)
Diabetes	612	27 (4.4)	4 (1.3)	23 (7.4)	940	52 (5.5)
Cardiovascular episode	613	1 (0.1)	0 (0.0)	1 (0.3)	940	14 (1.5)
Depression in lifetime	613	95 (15.5)	33 (11.0)	62 (19.8)	885	194 (21.9)
Anxiety/phobia in lifetime	612	114 (18.6)	34 (11.4)	80 (22.6)	885	202 (22.8)

*Note*: Values are mean ± SD, or *n*, percentage of participants in category. Cognitive scores are *z*‐scores with 95% CI. Cardiovascular episodes represent self‐reported previous occurrence of angina, stroke and myocardial infarction.

**FIGURE 1:**
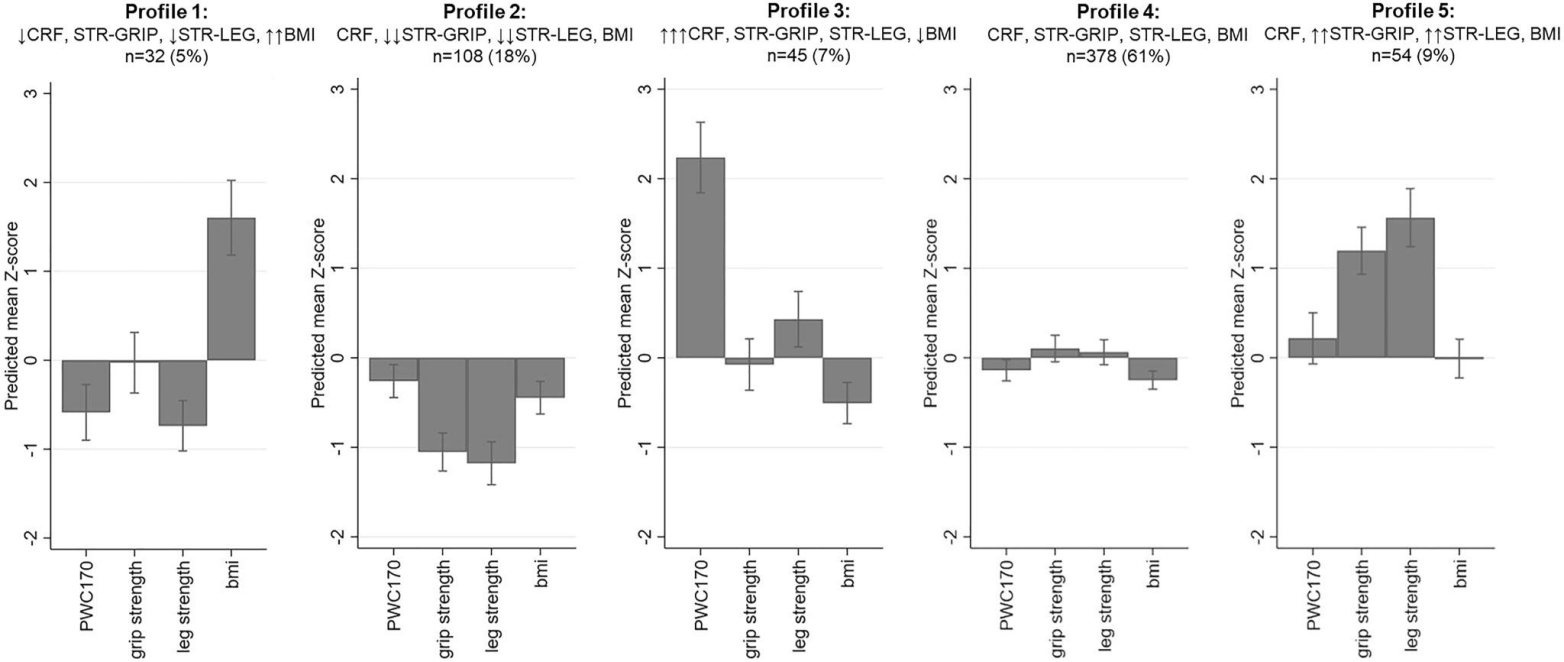
Mean age and sex adjusted *Z*‐scores for each midlife latent profile for total sample *n* = 617. A higher *Z*‐score for BMI is a worse outcome, for all other variables a higher *Z*‐score equates to greater performance. Profile 1: highest BMI/low fitness, profile 2: lowest muscular fitness/average CRF and BMI, profile 3: highest CRF fitness/average muscular fitness/low BMI, profile 4: average fitness and BMI, and profile 5: highest muscular fitness/average CRF and BMI. BMI, body mass index; CRF, cardiorespiratory fitness (PWC170: power output [watts] at a projected heart rate of 170 beats per minute); STR–GRIP, grip strength; STR–LEG, leg strength. ↑: 0.5–1 SD above average; ↑↑: >1–2 SD above average; ↑↑↑ >2 SD above average; ↓: 0.5–1 SD below average; ↓↓: >1–2 SD below average and ↓↓↓: >2 SD below average. No arrow: between 0 and 0.49 SD from average in either direction.

A five‐profile LPA model was the most appropriate fit (Figure S3). Participant characteristics for each profile are presented in Table S2. All profiles were well balanced between males and females, except for profile 3 which had a higher proportion of males. Mean *Z*‐scores for fitness and obesity measures are visualized in Figure 1.

**FIGURE 2:**
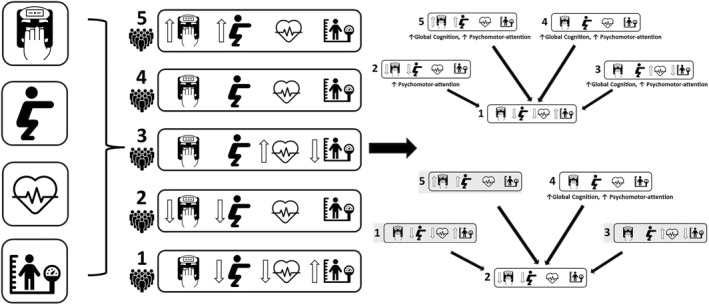
Visualization of the profiles and associations with cognitive performance where all other profiles were compared to profile 1 and profile 2. The right side of the diagram indicates associations between the reference profile and all other profiles; top: compared to reference profile 1; bottom: compared to reference profile 2. Arrows between profiles and the reference indicate a significant association was revealed. Shading indicates that no significant associations were detected.

Table 2 shows the associations between the different profiles and each cognitive composite. Compared to reference profile 1 (highest BMI/low fitness), profiles 3 (highest CRF/average muscular fitness/low BMI), 4 (average fitness and BMI) and 5 (highest muscular fitness/average CRF and BMI) were associated with higher scores on the global cognition and the psychomotor‐attention, and profile 2 (lowest muscular fitness/average CRF and BMI) was only associated with higher scores on the psychomotor‐attention composite. In addition, profile 2 (lowest muscular fitness/average CRF and BMI) was associated with lower global cognition [−0.17 (95% CI −0.34, −0.01), *p* = 0.040)] and psychomotor‐attention [−0.22 (95% CI −0.42, −0.03), *p* = 0.026)] compared to profile 4 (average fitness and BMI) in all models. There were no statistically significant associations with learning–working memory. The variables contributing to create the unique profiles, the profiles themselves and the results of analysis are visualized in Figure 2.

**TABLE 2:** Associations between midlife fitness and obesity profiles and midlife cognitive performance (*Z*‐scores; *N* = 617).

	Global cognition		Learning‐working memory		Psychomotor‐attention	
	β‐value (95% CI)		β‐value (95% CI)		β‐value (95% CI)	
Reference profile 1 [↓CRF, STR–GRIP, ↓STR–LEG, ↑↑BMI]	−0.43	(−0.74, −0.13)	−0.17	(−0.60, 0.26)	−0.52	(−0.89, −0.14)
Model 1		*n* = 617		*n* = 484		*n* = 613
Profile 2 [CRF, ↓↓STR–GRIP, ↓↓STR–LEG, BMI]	**0.31**	**(0.02, 0.61)**	0.13	(−0.26, 0.51)	**0.37**	**(0.02, 0.72)**
Profile 3 [↑↑↑CRF, STR‐GRIP, STR‐LEG, ↓BMI]	**0.60**	**(0.26, 0.94)**	0.24	(−0.19, 0.68)	**0.73**	**(0.33, 1.13)**
Profile 4 [CRF, STR–GRIP, STR–LEG, BMI]	**0.50**	**(0.23, 0.76)**	0.18	(−0.18, 0.53)	**0.61**	**(0.29, 0.93)**
Profile 5 [CRF, ↑↑STR–GRIP, ↑↑STR–LEG, BMI]	**0.45**	**(0.12, 0.77)**	0.07	(−0.36, 0.50)	**0.62**	**(0.23, 1.01)**
Model 2		*n* = 617		*n* = 484		*n* = 613
Profile 2 [CRF, ↓↓STR–GRIP, ↓↓STR–LEG, BMI]	**0.30**	**(0.01, 0.60)**	0.11	(−0.27, 0.50)	**0.36**	**(0.01, 0.71)**
Profile 3 [↑↑↑CRF, STR‐GRIP, STR‐LEG, ↓BMI]	**0.54**	**(0.20, 0.88)**	0.15	(−0.29, 0.58)	**0.68**	**(0.28, 1.09)**
Profile 4 [CRF, STR–GRIP, STR–LEG, BMI]	**0.48**	**(0.21, 0.75)**	0.15	(−0.21, 0.50)	**0.60**	**(0.28, 0.91)**
Profile 5 [CRF, ↑↑STR–GRIP, ↑↑STR–LEG, BMI]	**0.43**	**(0.11, 0.76)**	0.05	(−0.38, 0.48)	**0.61**	**(0.22, 0.99)**
Model 3		*n* = 617		*n* = 484		*n* = 613
Profile 2 [CRF, ↓↓STR–GRIP, ↓↓STR–LEG, BMI]	0.29	(−0.001, 0.58)	0.11	(−0.27, 0.49)	**0.35**	**(0.001, 0.70)**
Profile 3 [↑↑↑CRF, STR‐GRIP, STR‐LEG, ↓BMI]	**0.53**	**(0.19, 0.87)**	0.14	(−0.30, 0.57)	**0.68**	**(0.27, 1.08)**
Profile 4 [CRF, STR–GRIP, STR–LEG, BMI]	**0.47**	**(0.20, 0.74)**	0.15	(−0.21, 0.50)	**0.59**	**(0.27, 0.91)**
Profile 5 [CRF, ↑↑STR–GRIP, ↑↑STR–LEG, BMI]	**0.44**	**(0.12, 0.77)**	0.07	(−0.36, 0.50)	**0.62**	**(0.24, 1.01)**

*Note*: Model 1: unadjusted; Model 2: age at assessment, sex and education level. Model 3: adjusted for age, sex, education level, smoking history and alcohol consumption at adulthood with data imputed for alcohol (*n* = 5) and smoking (*n* = 36). Bolded values indicate significant associations between the profile and cognitive variables.

Abbreviations: BMI, body mass index; CRF, cardiorespiratory fitness (PWC170: power output (watts) at a projected heart rate of 170 beats per minute); STR–GRIP, grip strength; STR–LEG, leg strength.

Results of the univariate models showing the individual associations between standardized fitness and obesity variables and cognitive performance are presented in Table S3. Lower BMI and greater isometric grip and leg strength were all associated with higher scores in global cognition and psychomotor‐attention (all *p* < 0.05). After additional adjustment for smoking and alcohol consumption, lower BMI and greater grip strength were associated with better learning–working memory (both *p* < 0.05). Stepwise regression revealed that higher BMI was independently associated with poorer global cognition [−0.12 (95% CI −0.20, −0.04), *p* = 0.003)] and lower scores in psychomotor‐attention [−0.12 (95% CI −0.21, −0.02), *p* = 0.016)] after full adjustment for covariates.

## 4 DISCUSSION

In this study, midlife fitness and BMI were used to create distinct profiles, and their associations were examined with cognitive performance. Compared to a profile of low fitness and the highest BMI levels (profile 1), three profiles were associated with better scores on global cognitive and psychomotor‐attention measures. These profiles consisted of (1) average fitness and BMI (profile 4), (2) the highest CRF, average muscular fitness and low BMI (profile 3) and (3) the highest muscular fitness, average CRF and average BMI (profile 5). In addition, a profile consisting of the lowest muscular fitness, average CRF and average BMI (profile 2) was also associated with higher psychomotor‐attention. These associations were independent of potential confounding factors, including age, sex, educational attainment, smoking and alcohol consumption. No associations were found with a learning‐working memory composite. Our findings suggest that adults with good CRF and strength, and a healthy weight, appear to have better cognitive functioning and may be important targets, among other lifestyle factors, for preserving midlife cognition.

To our knowledge, this is the first study to demonstrate associations between phenotypic profiles of objectively measured fitness and obesity measured at midlife and their associations with different cognitive domains. Our analysis collectively extends evidence, whereby CRF (Tarumi et al., 2013) and obesity measures (Dahl et al., 2013; Smith et al., 2011) were independently associated with poorer midlife cognition. We observed that profile 1, characterized by the highest levels of BMI and low fitness, exhibited lower scores in psychomotor‐attention compared to all other profiles (profiles 2–5). Interestingly, even a profile with the lowest muscular fitness, but average CRF and slightly below average BMI (profile 2), had better psychomotor attention than profile 1, albeit associations were weaker than other profiles. Participants classified in profile 1 had above average levels of BMI (38.1 kg/m^2^) compared to the rest of the study sample with all individuals classified as obese. This suggests that a combination of obesity and low levels of fitness (profile 1) may be more detrimental to cognitive performance than poor fitness alone. The influence of BMI may also be highlighted in the stepwise regression models, where BMI remained as a significant predictor for the majority of cognitive measures despite the systematic addition of fitness variables. Our findings highlight that when designing interventions to preserve midlife cognition, it may be important to consider and target obesity and fitness levels together rather than separately. There is evidence that moderate to high CRF may counteract negative effects of obesity on health outcomes, according to the ‘fat but fit’ paradox (Ortega et al., 2018). For example, higher levels of CRF have attenuated the influence of obesity on cardiovascular disease and its risk factors in youth (DuBose et al., 2007). However, it remains uncertain if higher levels of CRF or muscular strength can mitigate the impact of high BMI on cognition, as a profile containing individuals with high BMI and high CRF did not emerge from the LPA.

Intriguingly, when examining associations between other profiles (2–5) and cognition, there were no differences, except that profile 4 (average fitness and BMI) was associated with higher global cognition and psychomotor attention compared to profile 2 (lowest muscular fitness/average CRF and BMI), supporting prior findings that have found associations between muscular fitness and cognition (Adamo et al., 2020; Liu et al., 2019). The non‐significant findings between other profiles (e.g., profile 2 (ref) and profiles 3 and 5; profile 3 (ref) and profiles 4 and 5; profile 4 (ref) and profile 5) are interesting as all had similar levels of BMI, adding weight to the importance of a healthy BMI for midlife cognition.

Interestingly, we only found associations between profiles of fitness and obesity with psychomotor‐attention and global cognition (most likely driven by psychomotor attention) but not learning‐working memory. Previous studies of participants in middle age found no differences in visual, episodic and delayed‐recall memory and learning when comparing the highest to lowest tertiles of obesity (Wolf et al., 2007), no differences in working memory when comparing highest to lowest tertiles of hand grip strength (Lu et al., 2021) and no association between grip strength and working memory (Adamo et al., 2020). Although these studies used different assessments of working memory and included some older participants (40–69 years), they partially support our null findings. In addition, this may have been due to the smaller sample size with this composite score; however, the standardized beta coefficients were also smaller than associations with other domains. Furthermore, working memory has been shown to decline in later life, whereas domains, such as processing speed, may start to decline after the age of 30 (Salthouse, 2012). Thus, in 36‐ to 49‐year‐olds, psychomotor speed and attention may already be exhibiting sub‐clinical changes, while working memory is intact.

Mechanisms underpinning the relationship between obesity, muscular and CRF with cognition are multifactorial. The relationship with obesity is likely to be largely driven by the associations with vascular disease (Kivipelto et al., 2005), underlying physical inactivity and the release of pro‐inflammatory markers and metabolites from excessive adipose tissue (Craft, 2009), which are all themselves linked to poorer cognitive health and performance. Conversely, higher CRF at midlife has been linked to better cognition through its associations with increased brain volume, synaptic plasticity and a reduction in vascular risk factors (Voss et al., 2013) and may promote similar benefits at midlife. Further mechanisms include neural growth factors, such as brain derived neurotrophic factor, which are released through aerobic exercise and resistance training. They are thought to mediate the beneficial effects of exercise on cognitive health and function as they are critical for neurogenesis, angiogenesis, and synaptic plasticity (Cotman et al., 2007). Aerobic exercise, and cardiovascular fitness, have also been reported to increase cerebral blood flow in humans and angiogenesis in the cortex and cerebellum in animal models (Stimpson et al., 2018). Regular exercise also increases the production of endogenous antioxidants, which have a role in reducing oxidative stress that can damage brain cells and impair cognitive function (Ionescu‐Tucker et al., 2021). As such, interventions, involving aerobic exercise (Smith et al., 2010) and intentional weight loss (Siervo et al., 2011), have produced modest improvements in domains including global cognition, psychomotor speed and attention in middle aged to older adults. Our findings also imply that higher levels of muscular strength are important for midlife cognition. This relationship is likely shaped by a combination of musculoskeletal factors (e.g., muscle mass) (Carson, 2018), neural mechanisms (e.g., common networks in pre motor cortex, cerebellum) and pathologies that overlap the coordination of strength and cognition (e.g., reductions in brain volume, greater white matter hyperintensity volume and subclinical vascular disease) (Debette et al., 2010). Given the potential impact of these mechanisms, strategies that focus on maintaining healthy weight and promoting strength and aerobic training should be promoted to preserve cognitive health and performance.

### 4.1 Limitations

Limitations of our study were that participants included in the analysis were relatively well‐educated, so the findings may not be generalizable to all Australian adults of the same age. Second, as this was a cross‐sectional analysis, and we have currently only measured cognitive performance at one timepoint, we cannot eliminate the possibility that a bidirectional relationship may exist, whereby cognitive performance drives BMI and fitness outcomes. Further, for CDAH‐3, there was missing data for fitness variables and the learning‐memory tests which may have reduced our ability to detect associations. Although there were few differences between those we included and participants not included in the analysis, it is possible that our findings may have differed had these variables been measured. Lastly, due to the nature of our LPA, our findings are limited to the profiles in our data, and therefore, we cannot make assumptions about other profiles that were not generated (e.g., high BMI and high CRF/muscular fitness). Our study had several strengths. To our knowledge, this is the first study to evaluate the associations of data‐driven profiles of fitness and obesity with cognitive function at midlife, including LPA and stepwise regression models. It included a well‐characterized national sample with ages younger than past studies and collected multiple objective measures rather than retrospective or self‐reported fitness data.

### 4.2 Conclusion

In summary, compared to the profile with low fitness levels and highest BMI, distinct profiles of higher and average fitness, and lower BMI, were associated with higher midlife psychomotor‐attention and global cognition but not learning memory. Higher CRF and strength, and a healthy weight, appear to be associated with better cognitive functioning and may be important targets, among other lifestyle factors, for preserving midlife cognition.

## CONFLICT OF INTEREST STATEMENT

There is no conflicts to declare.

## Supporting information

Supporting Information S1

## References

[ejsc12067-bib-0001] Adamo, Diane E. , Tara Anderson , Mahtab Koochaki , and Nora E. Fritz . 2020. “Declines in Grip Strength May Indicate Early Changes in Cognition in Healthy Middle‐Aged Adults.” PLoS One 15(4): e0232021. 10.1371/journal.pone.0232021. 32324794 PMC7179876

[ejsc12067-bib-0002] Brown, B. M. , J. J. Peiffer , and R. N. Martins . 2013. “Multiple Effects of Physical Activity on Molecular and Cognitive Signs of Brain Aging: Can Exercise Slow Neurodegeneration and Delay Alzheimer’s Disease?” Molecular Psychiatry 18(8): 864–874. 10.1038/mp.2012.162. 23164816

[ejsc12067-bib-0003] Carson, Richard G . 2018. “Get a Grip: Individual Variations in Grip Strength Are a Marker of Brain Health.” Neurobiology of Aging 71: 189–222. 10.1016/j.neurobiolaging.2018.07.023. 30172220

[ejsc12067-bib-0004] Clouston, S. A. P. , P. Brewster , D. Kuh , M. Richards , R. Cooper , R. Hardy , M. S. Rubin , and S. M. Hofer . 2013. “The Dynamic Relationship between Physical Function and Cognition in Longitudinal Aging Cohorts.” Epidemiologic Reviews 35(1): 33–50. 10.1093/epirev/mxs004. 23349427 PMC3578448

[ejsc12067-bib-0005] Cotman, Carl W. , Nicole C. Berchtold , and L.‐Ann Christie . 2007. “Exercise Builds Brain Health: Key Roles of Growth Factor Cascades and Inflammation.” Trends in Neurosciences 30(9): 464–472. 10.1016/j.tins.2007.06.011. 17765329

[ejsc12067-bib-0006] Craft, Suzanne . 2009. “The Role of Metabolic Disorders in Alzheimer Disease and Vascular Dementia: Two Roads Converged.” Archives of Neurology 66(3): 300–305. 10.1001/archneurol.2009.27. PMC2717716 19273747

[ejsc12067-bib-0007] Cui, Mengzhao , Siwen Zhang , Yujia Liu , Xiaokun Gang , and Guixia Wang . 2021. “Grip Strength and the Risk of Cognitive Decline and Dementia: A Systematic Review and Meta‐Analysis of Longitudinal Cohort Studies.” Frontiers in Aging Neuroscience 13: 1. 10.3389/fnagi.2021.625551. PMC7890203 33613270

[ejsc12067-bib-0008] Dahl, A. K. , L. B. Hassing , E. I. Fransson , M. Gatz , C. A. Reynolds , and N. L. Pedersen . 2013. “Body Mass Index across Midlife and Cognitive Change in Late Life.” International Journal of Obesity 37(2): 296–302. 10.1038/ijo.2012.37. 22450854 PMC3387354

[ejsc12067-bib-0009] Darby, D. G. , J. Fredrickson , R. H. Pietrzak , P. Maruff , M. Woodward , and A. Brodtmann . 2014. “Reliability and Usability of an Internet‐Based Computerized Cognitive Testing Battery in Community‐Dwelling Older People.” Computers in Human Behavior 30: 199–205. 10.1016/j.chb.2013.08.009.

[ejsc12067-bib-0010] Davies, Sasha J. , Jarrad A. G. Lum , Helen Skouteris , Linda K. Byrne , and Melissa J. Hayden . 2018. “Cognitive Impairment during Pregnancy: A Meta‐analysis.” Medical Journal of Australia 208(1): 35–40. 10.5694/mja17.00131. 29320671

[ejsc12067-bib-0011] Debette, S. , and H. S. Markus . 2010. “The Clinical Importance of White Matter Hyperintensities on Brain Magnetic Resonance Imaging: Systematic Review and Meta‐Analysis.” BMJ 341: c3666. 10.1136/bmj.c3666. 20660506 PMC2910261

[ejsc12067-bib-0012] DeFina, Laura F. , Benjamin L. Willis , Nina B. Radford , Ang Gao , David Leonard , William L. Haskell , Myron F. Weiner , and Jarett D. Berry . 2013. “The Association between Midlife Cardiorespiratory Fitness Levels and Later‐Life Dementia: A Cohort Study.” Annals of Internal Medicine 158(3): 162–168. 10.7326/0003-4819-158-3-201302050-00005. 23381040 PMC3926646

[ejsc12067-bib-0013] DuBose, Katrina D. , Joey C. Eisenmann , and Joseph E. Donnelly . 2007. “Aerobic Fitness Attenuates the Metabolic Syndrome Score in Normal‐Weight, At‐Risk‐For‐Overweight, and Overweight Children.” Pediatrics 120(5): e1262–e1268. 10.1542/peds.2007-0443. 17974719

[ejsc12067-bib-0014] Dwyer, T. , and L. E. Gibbons . 1994. “The Australian Schools Health and Fitness Survey. Physical Fitness Related to Blood Pressure but Not Lipoproteins.” Circulation 89(4): 1539–1544. 10.1161/01.cir.89.4.1539. 8149519

[ejsc12067-bib-0015] Fraser, Brooklyn J. , Quan L. Huynh , Michael D. Schmidt , Terence Dwyer , Alison J. Venn , and Costan G. Magnussen . 2016. “Childhood Muscular Fitness Phenotypes and Adult Metabolic Syndrome.” Medicine & Science in Sports & Exercise 48(9): 1715–1722. 10.1249/mss.0000000000000955. 27128670

[ejsc12067-bib-0016] Ionescu‐Tucker, Andra , and Carl W. Cotman . 2021. “Emerging Roles of Oxidative Stress in Brain Aging and Alzheimer's Disease.” Neurobiology of Aging 107: 86–95. 10.1016/j.neurobiolaging.2021.07.014. 34416493

[ejsc12067-bib-0017] Kivipelto, Miia , Tiia Ngandu , Laura Fratiglioni , Matti Viitanen , Ingemar Kåreholt , Bengt Winblad , E.‐Liisa Helkala , Jaakko Tuomilehto , Hilkka Soininen , and Aulikki Nissinen . 2005. “Obesity and Vascular Risk Factors at Midlife and the Risk of Dementia and Alzheimer Disease.” Archives of Neurology 62(10): 1556–1560. 10.1001/archneur.62.10.1556. 16216938

[ejsc12067-bib-0018] Knopman, David S. , Rebecca F. Gottesman , A. Richey Sharrett , Amanda L. Tapia , Sonia DavisThomas , B. Gwen Windham , Laura Coker , et al. 2018. “Midlife Vascular Risk Factors and Midlife Cognitive Status in Relation to Prevalence of Mild Cognitive Impairment and Dementia in Later Life: The Atherosclerosis Risk in Communities Study.” Alzheimer's and Dementia 14(11): 1406–1415. 10.1016/j.jalz.2018.03.011. PMC6231996 29763593

[ejsc12067-bib-0019] Lee, K. J. , and J. B. Carlin . 2010. “Multiple Imputation for Missing Data: Fully Conditional Specification versus Multivariate Normal Imputation.” American Journal of Epidemiology 171(5): 624–632. 10.1093/aje/kwp425. 20106935

[ejsc12067-bib-0020] Lim, Y. Y. , R. H. Pietrzak , P. Bourgeat , D. Ames , K. A. Ellis , A. Rembach , K. Harrington , et al. 2015. “Relationships between Performance on the Cogstate Brief Battery, Neurodegeneration, and Abeta Accumulation in Cognitively Normal Older Adults and Adults with MCI.” Archives of Clinical Neuropsychology 30(1): 49–58. 10.1093/arclin/acu068. 25467942

[ejsc12067-bib-0021] Liu, Yong , Xinyi Cao , Nannan Gu , Bixi Yang , Jijun Wang , and Chunbo Li . 2019. “A Prospective Study on the Association between Grip Strength and Cognitive Function Among Middle‐Aged and Elderly Chinese Participants.” Frontiers in Aging Neuroscience 11: 250. 10.3389/fnagi.2019.00250. 31551762 PMC6747049

[ejsc12067-bib-0022] Lopez‐Lopez, Jose P. , Daniel D. Cohen , Daniela Ney‐Salazar , Daniel Martinez , Johanna Otero , Diego Gomez‐Arbelaez , Paul A. Camacho , et al. 2021. “The Prediction of Metabolic Syndrome Alterations Is Improved by Combining Waist Circumference and Handgrip Strength Measurements Compared to Either Alone.” Cardiovascular Diabetology 20(1): 1–11. 10.1186/s12933-021-01256-z. 33752666 PMC7986558

[ejsc12067-bib-0023] Lu, Shenghua , Fabian Herold , Yanjie Zhang , Yuruo Lei , Arthur F. Kramer , Can Jiao , Qian Yu , et al. 2021. “Higher Handgrip Strength Is Linked to Better Cognitive Performance in Chinese Adults with Hypertension.” Brain Sciences 11(8): 985. 10.3390/brainsci11080985. 34439604 PMC8391417

[ejsc12067-bib-0024] Malmstrom, Theodore K. , Fredric D. Wolinsky , Elena M. Andresen , J. Philip Miller , and Douglas K. Miller . 2005. “Cognitive Ability and Physical Performance in Middle‐aged African Americans.” Journal of the American Geriatrics Society 53(6): 997–1001. 10.1111/j.1532-5415.2005.53318.x. 15935023

[ejsc12067-bib-0025] Maruff, Paul , Yen Ying Lim , David Darby , Kathryn A. Ellis , Robert H. Pietrzak , Peter J. Snyder , Ashley I. Bush , et al. 2013. “Clinical Utility of the Cogstate Brief Battery in Identifying Cognitive Impairment in Mild Cognitive Impairment and Alzheimer's Disease.” BMC Psychology 1(1): 30. 10.1186/2050-7283-1-30. 25566378 PMC4269990

[ejsc12067-bib-0026] Mekari, Said , Olivier Dupuy , Ricardo Martins , Kailey Evans , Derek S. Kimmerly , Sarah Fraser , and Heather F. Neyedli . 2019. “The Effects of Cardiorespiratory Fitness on Executive Function and Prefrontal Oxygenation in Older Adults.” Geroscience 41(5): 681–690. 10.1007/s11357-019-00128-5. 31728899 PMC6885073

[ejsc12067-bib-0027] Oberski, D . 2016. “Mixture Models: Latent Profile and Latent Class Analysis.” In Modern Statistical Methods for HCI. Springer.

[ejsc12067-bib-0028] Ortega, Francisco B. , Jonatan R. Ruiz , Idoia Labayen , Carl J. Lavie , and Steven N. Blair . 2018. “The Fat but Fit Paradox: What We Know and Don’t Know about it.” British Journal of Sports Medicine 52(3): 151–153. 10.1136/bjsports-2016-097400. 28583992

[ejsc12067-bib-0029] Pedersen, Maria , Karin Kaereby Pedersen , Helle Bruunsgaard , Karen Suarez Krabbe , Carsten Thomsen , Kristine Færch , Bente Klarlund Pedersen , and Erik Lykke Mortensen . 2012. “Cognitive Functions in Middle Aged Individuals Are Related to Metabolic Disturbances and Aerobic Capacity: A Cross‐Sectional Study.” PLoS One 7(12): e51132. 10.1371/journal.pone.0051132. 23251434 PMC3521021

[ejsc12067-bib-0030] Peng, T.‐Chun , W.‐Liang Chen , Li‐Wei Wu , Y.‐Wen Chang , and T.‐Wei Kao . 2020. “Sarcopenia and Cognitive Impairment: A Systematic Review and Meta‐Analysis.” Clinical Nutrition 39(9): 2695–2701. 10.1016/j.clnu.2019.12.014. 31917049

[ejsc12067-bib-0031] Rovio, Suvi P. , Katja Pahkala , Jaakko Nevalainen , Markus Juonala , Pia Salo , Mika Kähönen , Nina Hutri‐Kähönen , et al. 2016. “Cognitive Performance in Young Adulthood and Midlife: Relations with Age, Sex, and Education—The Cardiovascular Risk in Young Finns Study.” Neuropsychology 30(5): 532–542. 10.1037/neu0000239. 26523520

[ejsc12067-bib-0032] Salthouse, Timothy . 2012. “Consequences of Age‐Related Cognitive Declines.” Annual Review of Psychology 63(1): 201–226. 10.1146/annurev-psych-120710-100328. PMC3632788 21740223

[ejsc12067-bib-0033] Siervo, M. , R. Arnold , J. C. K. Wells , A. Tagliabue , A. Colantuoni , E. Albanese , C. Brayne , and B. C. M. Stephan . 2011. “Intentional Weight Loss in Overweight and Obese Individuals and Cognitive Function: A Systematic Review and Meta‐analysis.” Obesity Reviews 12(11): 968–983. 10.1111/j.1467-789x.2011.00903.x. 21762426

[ejsc12067-bib-0034] Smith, E. , P. Hay , L. Campbell , and J. N. Trollor . 2011. “A Review of the Association between Obesity and Cognitive Function across the Lifespan: Implications for Novel Approaches to Prevention and Treatment.” Obesity Reviews 12(9): 740–755. 10.1111/j.1467-789x.2011.00920.x. 21991597

[ejsc12067-bib-0035] Smith, Patrick J. , James A. Blumenthal , Benson M. Hoffman , Harris Cooper , Timothy A. Strauman , Kathleen Welsh‐Bohmer , Jeffrey N. Browndyke , and Andrew Sherwood . 2010. “Aerobic Exercise and Neurocognitive Performance: A Meta‐Analytic Review of Randomized Controlled Trials.” Psychosomatic Medicine 72(3): 239–252. 10.1097/psy.0b013e3181d14633. 20223924 PMC2897704

[ejsc12067-bib-0036] Stimpson, Nikolas J. , Glen Davison , and Amir‐Homayoun Javadi . 2018. “Joggin’the Noggin: Towards a Physiological Understanding of Exercise‐Induced Cognitive Benefits.” Neuroscience & Biobehavioral Reviews 88: 177–186. 10.1016/j.neubiorev.2018.03.018. 29572187

[ejsc12067-bib-0037] Tarumi, Takashi , Mitzi M. Gonzales , Bennett Fallow , Nantinee Nualnim , Jeongseok Lee , Hirofumi Tanaka , and Andreana P. Haley . 2013. “Aerobic Fitness and Cognitive Function in Midlife: An Association Mediated by Plasma Insulin.” Metabolic Brain Disease 28(4): 727–730. 10.1007/s11011-013-9431-1. 24000071 PMC4394746

[ejsc12067-bib-0038] Voss, Michelle W. , Carmen Vivar , Arthur F. Kramer , and Henriette van Praag . 2013. “Bridging Animal and Human Models of Exercise‐Induced Brain Plasticity.” Trends in Cognitive Sciences 17(10): 525–544. 10.1016/j.tics.2013.08.001. 24029446 PMC4565723

[ejsc12067-bib-0039] Wolf, Philip , Alexa Beiser , Merrill Elias , Rhoda Au , Ramachandran Vasan , and Sudha Seshadri . 2007. “Relation of Obesity to Cognitive Function: Importance of Central Obesity and Synergistic Influence of Concomitant Hypertension. The Framingham Heart Study.” Current Alzheimer Research 4(2): 111–116. 10.2174/156720507780362263. 17430232

[ejsc12067-bib-0040] World Health Organization . 1997. Composite International Diagnostic Interview, CIDI‐Auto 2.1: Administrator’s Guide and Reference. Geneva: World Health Organization.

