# Supplementary material for: Associations of midlife fitness and obesity profiles with cognitive function
Source: Eur J Sport Sci. 2024 Mar 18;24(5):587–96. doi: 10.1002/ejsc.12067 (PMC11235945; doi:10.1002/ejsc.12067)
Supplement: Supplementary file 1 — Supporting Information S1 [file EJSC-24-587-s001.docx]

Supplementary Table 1: Description of the cognitive tasks used in the CogState computerized test battery.

| **Test name (Abbreviation)** | **Cognitive**  **domain** | **Outcome**  **measure** | **Task Completion Criteria** | **Task Instructions** |
| --- | --- | --- | --- | --- |
| Detection Task | Simple reaction time, speed of processing | Mean of the log 10 transformed reaction times for correct responses (Log_10_ milliseconds) | At least 27 responses made | A generic joker card is presented face down in the centre of the computer screen. When this card flips over participants are required to press the “Yes” key (denoted as K on the keyboard) as quickly as possible |
| Identification Task | Choice reaction time, visual attention | Mean of the log 10 transformed reaction times for correct responses (Log_10_ milliseconds) | At least 23 responses made | A playing card is shown face down in the centre of the screen; when the card flips over to reveal the picture (either a red joker or black joker), the participant is required to indicate the colour of the joker in response to the question “Is the card red”?, pressing the “Yes” or “No” key (D key on the keyboard) |
| One Card Learning Task | Visual memory, attention | Arcsine transformed correct responses/total responses | At least 66 responses made | A playing card is shown face down in the centre of the screen. When the card flips over, participants must indicate as quickly and accurately as possible whether they have seen that card previously in this task, pressing either “Yes” or “No” keys. |
| One Back Task | Working memory, attention | Mean of the log 10 transformed reaction times for correct responses (Log_10_ milliseconds) | At least 24 responses made | A playing card is shown face down and when it flips over, participants must decide whether the card displayed is exactly the same (same suit and same value) as the card presented immediately prior, responding as quickly as possible using “Yes” or “No” keys. |

**Supplementary Fig. 1** Summary of the statistical analyses.

**Latent Profile Analysis**

**n=617**

Midlife: Muscular fitness variables:

- PWC170
- leg strength
- grip strength
- body mass index
- sex

**Estimation of latent profile models ranging from**

**2 to 10 profiles:**

- Akaike information Criterion and Bayesian information criterion
- Elbow point in scree plot of model log-likelihood
- Profile membership n≥5
- Clinical usefulness and interpretability
- Midlife individual cognitive scores standardised to create Z-scores
- Composites created from Z-scores:
- Global Cognitive function
- Learning-Working Memory
- Psychomotor-Attention

**Standardised midlife fitness variables:**

- PWC170
- leg strength
- grip strength
- body mass index

**Linear Regression of standardised fitness variables with:**

- Global Cognitive function
- Learning-Working Memory Composite
- Psychomotor-Attention Composite
  - Adjusted for age, sex, education level, smoking, alcohol

**Linear Regression of latent profile analysis with:**

- Global Cognitive function
- Learning-Working Memory Composite
- Psychomotor-Attention Composite
  - Adjusted for age, sex, smoking, alcohol, education

**Stepwise Linear Regression of standardised fitness variables with:**

- Global Cognitive function
- Learning-Working Memory Composite
- Psychomotor-Attention Composite
  - Adjusted for age, sex, smoking, alcohol, education

PWC170: power output (watts) at a projected heart rate of 170 beats per minute

**Supplementary Fig. 2** Flow diagram of participants through the study.

Participants aged 7-15 years

n=8498

Attended CDAH-3 clinic aged 36-49 years

n=1567

Did not complete cognitive testing due to time constraints, medical episodes, refused or opted for shorter protocol

n=276

Of these n=276, did not complete all physical testing due to time constraints, medical episode during testing (e.g., increased BP), injured or shorter protocol

N=137

Completed cognitive assessment

n=1291

Participants with cognitive data

n=1244

Participants that did not satisfy requirements for any task (n=47)

Individual tests failing task criteria:

- DET: n=123
- IDN: n=64
- ONB: n=342
- OCL: n=19

Missing data:

- grip strength: n=299
- leg strength: n=343
- PWC170: n=570
- body mass index: n=5

Pregnant women n=3

Participants with at least one cognitive test and all midlife fitness and obesity measures

n=617

CDAH-3: Childhood Determinants of Health Study follow up 3, DET: Detection task, IDN: Identification task, OCL: One Card Learning task, ONB: One Back task**,** PWC170: power output (watts) at a projected heart rate of 170 beats per minute

**Supplementary Table 2:** Mean scores for midlife profiles (n=617).

|  | | **Profile 1**  ↓CRF, STR ̶ GRIP,  ↓STR ̶ LEG, ↑↑BMI  **n=32**  Mean (SD; range) | | | **Profile 2**  CRF, ↓↓STR ̶ GRIP,  ↓↓STR ̶ LEG, BMI  **n=108**  Mean (SD; range) | | | | **Profile 3**  ↑↑↑CRF, STR ̶ GRIP, STR ̶ LEG, ↓BMI  **n=45**  Mean (SD; range) | | | | **Profile 4**  CRF, STR ̶ GRIP,  STR ̶ LEG, BMI  **n=378**  Mean (SD; range) | | | | **Profile 5**  CRF, ↑↑STR ̶ GRIP,  ↑↑STR ̶ LEG, BMI  **n=54**  Mean (SD; range) | | | | |
| --- | --- | --- | --- | --- | --- | --- | --- | --- | --- | --- | --- | --- | --- | --- | --- | --- | --- | --- | --- | --- | --- |
| Females, n (%) | | 19/32 | | | 63/108 | | | | 13/45 | | | | 196/378 | | | | 23/54 | | | | |
| Age (years) | 44.8 | | (2.5; 39.7 ̶ 48.6) | | | 45.0 | (2.6; 39.6 ̶ 49.1) | | | 44.1 | (2.1; 40.7 ̶ 48.1) | | | 44.5 | (2.6; 39.3 ̶ 49.5) | | 44.4 | | (2.6; 39.5 ̶ 49.0) | |  |
| Weight (kg) | 109.6 | | (12.4; 87.4 ̶ 140.4) | | | 71.6 | (13.9; 40.7 ̶ 108.7) | | | 78.2 | (15.2; 52.3 ̶ 113.7) | | | 78.5 | (14.3; 46.3 ̶ 120.2) | | 86.5 | | (16.7; 53.8 ̶ 130.0) | |  |
| Height (cm) | 169.8 | | (8.9; 150.5 ̶ 191.8) | | | 169.2 | (9.6; 145.9 ̶ 188.9) | | | 176.5 | (9.1; 158.2 ̶ 198.6) | | | 172.8 | (9.2; 148.2 ̶ 196.1) | | 175.8 | | (9.5; 156.5 ̶ 194.6) | |  |
| BMI (kg/m^2^) | 38.1 | | (3.8; 33.0 ̶ 48.9) | | | 24.9 | (3.9; 17.6 ̶ 33.6) | | | 24.9 | (3.3; 18.2 ̶ 32.7) | | | 26.2 | (3.7; 16.9 ̶ 35.2) | | 27.9 | | (4.4; 19.8 ̶ 40.4) | |  |
| Education- university | 43.8% | | |  | | 53.7% | |  | | 71.1% | |  | | 55.6 % | |  | | 48.2 % | |  |  |
| Hypertension | 21.9% | | |  | | 10.3% | |  | | 4.4% | |  | | 10.7% | |  | | 13.0% | |  |  |
| Diabetes | 12.5 % | | |  | | 3.7 % | |  | | 0 % | |  | | 5.1 % | |  | | 0 % | |  |  |
| Diagnosis of depression | 25.8 % | | |  | | 15.0 % | |  | | 17.8 % | |  | | 15.2 % | |  | | 11.1 % | |  |  |
| Diagnosis of anxiety | 25.8 % | | |  | | 20.8 % | |  | | 13.3 % | |  | | 17.6 % | |  | | 22.2 % | |  |  |

BMI: body mass index, CRF: cardiorespiratory fitness [PWC170- power output (watts) at a projected heart rate of 170 beats per minute], STR ̶ GRIP: grip strength, STR ̶ LEG: leg strength. Z-scores in profiles were labelled as follows; ↑: 0.5 ̶ 1 SD above average; ↑↑: >1 ̶ 2 SD above average; ↑↑↑ >2 SD above average; ↓: 0.5 ̶ 1 SD below average; ↓↓: >1 ̶ 2 SD below average; ↓↓↓: >2 SD below average. No arrow: between 0 and 0.49 SD from average in either direction. Range: minimum to maximum values for each profile.

**Supplementary Table 3:** Associations between standardised midlife fitness and obesity variables and midlife cognitive performance (Z-scores).

|  | **n** | **Global Cognition**  β-value (95% CI) | | **n** | **Learning-Working memory**  β-value (95% CI) | | **n** | **Psychomotor-attention**  β-value (95% CI) | |
| --- | --- | --- | --- | --- | --- | --- | --- | --- | --- |
| Model 1 |  |  |  |  |  |  |  |  |  |
| Grip strength (kg) | 617 | **0.06** | **(0.004, 0.13)** | 484 | 0.04 | (-0.03, 0.12) | 613 | **0.08** | **(0.01, 0.15)** |
| Leg strength (kg) | 617 | **0.08** | **(0.02, 0.14)** | 484 | 0.003 | (-0.07, 0.08) | 613 | **0.12** | **(0.05, 0.19)** |
| PWC170 (W/kg) | 617 | 0.02 | (-0.04, 0.08) | 484 | -0.01 | (-0.09, 0.06) | 613 | 0.04 | (-0.03, 0.11) |
| BMI | 617 | **-0.12** | **(-0.19, -0.05)** | 484 | -0.08 | (-0.17, 0.01) | 613 | **-0.11** | **(-0.20, -0.03)** |
| Model 2 |  |  |  |  |  |  |  |  |  |
| Grip strength (kg) | 617 | **0.08** | **(0.03, 0.13)** | 484 | **0.09** | **(0.02, 0.15)** | 613 | **0.08** | **(0.03, 0.15)** |
| Leg strength (kg) | 617 | **0.09** | **(0.05, 0.14)** | 484 | 0.04 | (-0.03, 0.10) | 613 | **0.12** | **(0.06, 0.18)** |
| PWC170 (W/kg) | 617 | 0.03 | (-0.03, 0.09) | 484 | -0.01 | (-0.08, 0.07) | 613 | 0.05 | (-0.02, 0.12) |
| BMI | 617 | **-0.14** | **(-0.20, -0.09)** | 484 | **-0.10** | **(-0.17, -0.03)** | 613 | **-0.15** | **(-0.21, -0.08)** |

Model 1: adjusted for age, sex, education level, Model 2: additionally adjusted for smoking history and alcohol consumption- missing data imputed. BMI: body mass index, PWC170: power output (watts) at a projected heart rate of 170 beats per minute. Bolded values represent significant associations.

**Supplementary material (methods):**

Latent profile analysis (LPA) was used to identify childhood fitness and obesity profiles present. LPA identifies mutually exclusive classes which display characteristic patterns across included variables, by maximizing between-group variance and minimizing within-group variance^1^. Latent profiles were operationalised as independent variables by allocating each participant to profile most likely given their fitness and WHR data. We estimated models with 2-10 profiles using the Akaike and Bayesian information criteria^2^, a scree-plot of each model’s log-likelihood^3^ and clinical interpretability to decide which models best fit the data. Any model with one or more profile sizes of n ≤ 2 were ruled out, as this could indicate over-extraction^4^.

**
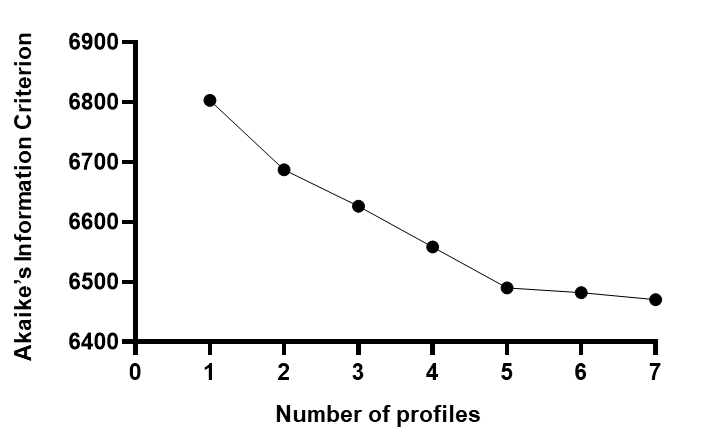
**

**Supplementary Fig. 3** Scree plot with estimates of Akaike’s information criterion by number of profiles generated by latent profile analysis. Elbow is visible at n=5

References:

1. Oberski D. Mixture models: Latent profile and latent class analysis, in Modern statistical methods for HCI, Springer, 2016.

2. Olivera-Aguilar M, Rikoon SH. Assessing measurement invariance in multiple-group latent profile analysis. *Structural Equation Modeling: A Multidisciplinary Journal.* 2018; 25(3):439-452.

3. Nylund KL, Asparouhov T, Muthén BO. Deciding on the number of classes in latent class analysis and growth mixture modeling: A Monte Carlo simulation study. *Structural equation modeling: A multidisciplinary Journal.* 2007; 14(4):535-569.

4. Lubke G, Neale MC. Distinguishing between latent classes and continuous factors: Resolution by maximum likelihood? *Multivariate Behavioral Research.* 2006; 41(4):499-532.
